# Immunadsorption bei rheumatischen Autoimmunerkrankungen: eine alternative Therapieoption: Retrospektive Erkenntnisse zur klinischen Anwendung aus dem Rheumazentrum Rheinland-Pfalz

**DOI:** 10.1007/s00393-025-01677-1

**Published:** 2025-07-22

**Authors:** Konstantinos Triantafyllias, Ann-Kathrin Dapper, Andreas Schwarting

**Affiliations:** 1 Rheumazentrum Rheinland-Pfalz, Bad Kreuznach, Deutschland; 2 Schwerpunkt Rheumatologie und klinische Immunologie, I. Med. Klinik und Poliklinik, Universitätsmedizin Mainz, Johannes Gutenberg-Universität, Langenbeckstr. 1, 55131 Mainz, Deutschland; 3 Klinik und Poliklinik für Neuroradiologie, Universitätsmedizin Mainz, Johannes Gutenberg-Universität, Mainz, Deutschland

**Keywords:** Systemisch entzündliche rheumatische Erkrankungen, Sicherheitsprofil, Bridging-Therapie, Immunglobulinspiegel, Individualisierte Therapieansätze, Systemic inflammatory rheumatic diseases, Safety profile, Bridging therapy, Immunoglobulin level, Individualized treatment approaches

## Abstract

**Hintergrund:**

Systemisch entzündliche rheumatische Erkrankungen gehen häufig mit einer hohen Morbidität und teils schweren chronischen Verläufen einher. Sie erfordern frühzeitige und effektive Behandlungsstrategien, um irreversible Schäden zu verhindern. Eine medikamentöse Immunsuppression ist nicht bei allen Patient:innen möglich. In solchen Fällen ist die Anwendung alternativer Therapien wie der Immunadsorption (IA) von besonderem Interesse. Trotz positiver klinischer Erfahrungen ist die Datenlage zur Anwendung der IA bei rheumatischen Erkrankungen begrenzt.

**Ziel der Arbeit:**

Ziel war die Analyse von Anwendung, Wirksamkeit und Sicherheit der IA unter Alltagsbedingungen.

**Material und Methoden:**

Im Rahmen dieser retrospektiven Untersuchung wurden 373 Immunadsorptionsbehandlungen (IA) bei 31 Patient:innen mit verschiedenen rheumatischen Erkrankungen durchgeführt.

**Ergebnisse und Diskussion:**

Die Ergebnisse zeigen, dass die IA überwiegend in Kombination mit anderen medikamentösen Verfahren eingesetzt wurde – häufig als „Bridging-Therapie“ oder Ultima Ratio in komplexen Fällen. Die Behandlung zeigte ein gutes Sicherheitsprofil; bei 25 % der Patient:innen traten vorwiegend milde Nebenwirkungen wie Blutdruckabfälle auf. Die IA führte zu einer signifikanten Reduktion der Immunglobulinspiegel. In 48 % der Fälle konnte ein positiver klinischer Therapieeffekt beobachtet werden. Kürzere Intervalle zwischen den Behandlungen waren mit einem besseren Ansprechen assoziiert (*p* = 0,004). Ein Zusammenhang zwischen einem positiven Therapieeffekt und der begleitenden Glukokortikoidtherapie konnte nicht festgestellt werden (*p* = 0,611), was auf einen potenziell eigenständigen Effekt der IA hinweist.

**Schlussfolgerung:**

Die IA kann somit eine wertvolle Ergänzung im Rahmen individualisierter Therapieansätze darstellen und sollte in zukünftigen Studien weiter evaluiert werden.

## Abstract (en)

**Background:**

Systemic inflammatory rheumatic diseases are frequently linked to significant morbidity and, in some cases, can lead to severe, chronic disease courses. Early and effective treatment strategies are required to prevent irreversible damage. Drug-based immunosuppression is not possible for all patients. In such cases the use of alternative treatment, such as immunoadsorption (IA) is of particular interest. Despite positive clinical experiences, the data on the use of IA in rheumatic diseases are limited.

**Objective:**

The aim was to analyze the application, efficacy and safety of IA under routine conditions.

**Material and methods:**

In this retrospective study immunoadsorption (IA) treatment was performed 373 times in 31 patients with various rheumatic diseases.

**Results and discussion:**

The results show that IA was predominantly used in combination with other medicinal procedures, often as a bridging therapy or as a last resort in complex cases. The treatment showed a good safety profile: 25% of patients experienced mainly mild side effects such as a short-term drops in blood pressure. The use of IA led to a significant reduction in immunoglobulin levels. A positive clinical therapeutic effect was observed in 48% of the cases. Shorter intervals between treatments were associated with a better clinical response (*p* = 0.004). A correlation between a positive therapeutic effect and the concomitant glucocorticoid therapy could not be established (*p* = 0.611), which could potentially indicate an independent effect of IA.

**Conclusion:**

The use of IA can be a valuable addition to individualized treatment approaches and should be further evaluated in future studies.

Systemisch entzündliche rheumatische Erkrankungen, oft durch Autoantikörper induziert, zeigen ein breites Spektrum akuter und chronischer Manifestationen [1, 2]. Besonders häufig ist der Bewegungsapparat betroffen, was zu Schmerzen, Funktionseinschränkungen und Gelenkdestruktionen führen kann [2]. Diese Erkrankungen sind mit erhöhter Morbidität und Mortalität (0,47 % aller Todesfälle) assoziiert [1, 3]. Die häufigste entzündliche Gelenkerkrankung ist die rheumatoide Arthritis (RA), von der 2019 weltweit 18 Mio. Menschen betroffen waren [4].

Ziel der Therapie ist die Reduktion der Entzündungsaktivität, Vermeidung irreversibler Schäden und Erhalt der Funktionalität [2, 5]. Eine frühzeitige Behandlung verbessert die Prognose und das Ansprechen auf die Therapie [2]. Häufig erfolgt die Behandlung mit Immunsuppressiva [6, 7]. Diese sind jedoch nicht für alle Patient:innen geeignet – etwa bei Immundefekten, geplanten Operationen, Schwangerschaft, Krebserkrankungen, Infektionen oder Allergien [8–10].

Die Immunadsorption (IA) ist ein Verfahren zur selektiven Entfernung bestimmter Plasmaproteine, das zur Modulation autoimmunologischer Prozesse eingesetzt wird [11–13]. Sie kann die Krankheitsaktivität senken, die Wirksamkeit anderer Therapien verbessern und die Medikamentendosis reduzieren [1, 11, 14]. Zudem hat sie gegenüber anderen Therapien Vorteile in Bezug auf allergische Reaktionen und Infektionen und weist ein geringes Potenzial für Nebenwirkungen auf [24]. Die IA hat sich in Studien als sicher und wirksam bei refraktärer RA und anderen Autoimmunerkrankungen erwiesen [12, 13], auch wenn belastbare Daten, insbesondere aus der Biologikaära, fehlen [11]. Poullin et al. plädieren daher dafür, ihren Einsatz nicht nur auf austherapierte Patient:innen zu beschränken [14].

Unsere retrospektive Analyse untersucht das Potenzial der IA als ergänzende oder alternative Therapie bei rheumatischen Erkrankungen. Dabei analysieren wir Häufigkeit, Wirksamkeit, Sicherheit sowie Begleittherapien und Behandlungsschemata in einem großen medizinischen Zentrum im klinischen Alltag.

## Methoden

### Studienpopulation

**Abb. 1:**
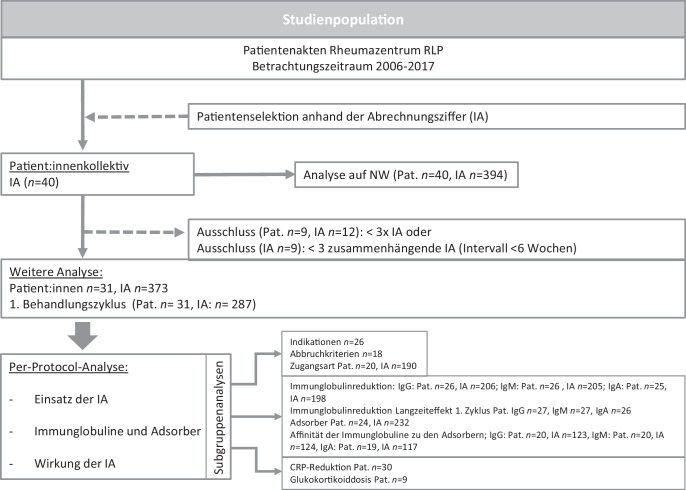
Studienpopulation. *RLP* Rheinland-Pfalz, *IA* Immunadsorption, *NW* Nebenwirkungen, *Pat.* Patienten, *Ig* Immunglobulin, *CRP* C-reaktives Protein

In unsere retrospektive Analyse wurden 40 Patient:innen mit rheumatischen Erkrankungen eingeschlossen, die zwischen 2006 und 2017 im Rheumazentrum Rheinland-Pfalz eine Immunadsorptionsbehandlung erhielten. Ausgeschlossen wurden Patient:innen unter 18 Jahren sowie solche mit weniger als 3 aufeinanderfolgenden IA-Sitzungen. Die Per-Protocol-Analyse umfasste 31 Patient:innen (Abb. 1).

### Datenerfassung

Retrospektiv erhoben wurden epidemiologische Daten, Informationen zur Behandlung, verwendete IA-Säulen sowie Laborwerte vor und nach der Therapie. Ein positiver Therapieeffekt wurde angenommen bei CRP(C-reaktives Protein)-Reduktion ohne Erhöhung der Glukokortikoiddosis und ohne Einleitung neuer immunsuppressiver Therapien. Bei CRP-Erhöhung, Dosissteigerung oder Therapieabbruchkriterien, die gegen einen Therapieerfolg sprachen, wurde kein positiver Therapieeffekt angenommen.

### Details der Immunadsorption

Die IA wurde stationär oder ambulant im Rheumazentrum Rheinland-Pfalz mit dem Gerät Octo Nova (Diamed Medizintechnik GmbH, Stadtwaldgürtel 77, 50935 Köln) durch eine nicht verblindete Immunadsorptionsfachkraft durchgeführt.

### Statistik

Die Normalverteilung wurde mittels Shapiro-Wilk-Test und grafisch (Quantile-Quantile-Plot) geprüft. Für Zusammenhänge zwischen nominalen Variablen wurden Chi-Quadrat- und Fisher’s Exact-Test verwendet. Abhängige metrische Variablen wurden mit t‑Test oder Wilcoxon-Test geprüft. Unabhängige Stichproben wurden je nach Verteilung mit t‑Test, ANOVA („analysis of variance“), Mann-Whitney-U- oder Kruskal-Wallis-Test analysiert. Ein *p*-Wert ≤0,05 galt als signifikant. Die Auswertung erfolgte mit IBM SPSS Statistics 23.0 (IBM Deutschland GmbH, IBM-Allee 1, 71139 Ehningen, Deutschland).

## Ergebnisse

In der Exploration wurden 373 Immunadsorptionsbehandlungen an 31 Patient:innen mit 3 verschiedenen Adsorbern – Globaffin (Fresenius Medical Care AG, Hof an der Saale, Deutschland), Tryptophan (DIAMED Medizintechnik GmbH, Köln, Deutschland) und Immunosorba (Fresenius Medical Care AG, Hof an der Saale, Deutschland) – durchgeführt. Die Patient:innen (52 % Frauen und 48 % Männer, Alter: Median = 50 Jahre, IQR [„interquartile range“]: 45–65) litten an verschiedenen rheumatische Erkrankungen (71 % rheumatoide Arthritis, 26 % Kollagenose/Vaskulitis, 3 % Spondyloarthritiden). Die Behandlungsanzahl variierte zwischen 3 und 54 (Median: 7, IQR: 4–14), die mittlere Behandlungsdauer betrug 48 Tage (IQR: 15–124) mit einem Behandlungsintervall von durchschnittlich 7 Tagen (IQR: 4,25–9,25). Sieben Patient:innen erhielten bis zu 4 Behandlungszyklen (≤ 6 Wochen Abstand zwischen den Sitzungen), insgesamt wurden 42 Zyklen dokumentiert. Ein Zyklus dauerte im Mittel 48 Tage. Die Details der Studienpopulation sind in Tab. 1 aufgeführt.

**Tab. 1:** Deskriptive Daten gesamte Studienpopulation

Charakteristika	Median (IQR)	*n*	Signifikanz
Alter (Jahre)	50 (45–65)	31	–
Geschlecht (weiblich)	52 %	31	–
Pos. Therapieeffekt ges.	48 %	31	–
Pos. Therapieeffekt ges. mit Glukokortikoiden (GK)	29 %	31	0,611
Pos. Therapieeffekt ges. ohne Glukokortikoide (GK)	19 %	31	
Pos. Therapieeffekt 1. Zyklus	48 %	31	–
Pos. Therapieeffekt 1. Zyklus mit GK	29 %	31	–
Pos. Therapieeffekt 1. Zyklus ohne GK	19 %	31	–
Anzahl der Behandlungen mittels IA ges.	7 (4–14)	31	–
Behandlungszeitraum (Tage) ges.	48 (15–124)	31	–
Behandlungsintervall (Tage) ges.	7 (4–9)	331	–
Erkrankungsdauer (Jahre)	6 (1–11)	21	–
Rheumafaktor positiv	90 %	31	–
Anti-CCP-positiv	39 %	31	–
CRP (mg/dl) *vor* Therapie	2,38 (0,93–4,87)	30	0,495
CRP (mg/dl) *nach* Therapie	1,79 (0,62–4,62)	30	
Immunsuppressiva	–	–	–
DMARD	32 %	31	–
Glukokortikoidgabe	65 %	31	–
Glukokortikoiddosis (mg) *vor* Therapie	15 (12,5–35)	9	0,715
Glukokortikoiddosis (mg) *nach* Therapie	12,5 (10–25)	9	
Schmerzmittel	65 %	31	–
Rheumatoide Arthritis	71 %	31	–
Kollagenosen/Vaskulitis	26 %	31	–
Spondylarthritiden	3 %	31	–
IgG-Reduktion (%) durch IA	−50 (−63 bis −38)	202	<0,001**
IgM-Reduktion (%) durch IA	−26 (−36 bis −16)	201	<0,001**
IgA-Reduktion (%) durch IA	−20 (−29 bis −15)	194	<0,001**
IgG *vor* Therapie (mg/dl)	1810 (1045–1965)	11	0,007**
IgG *nach* Therapie (mg/dl)	1070 (835–1526)	11	
IgM *vor* Therapie (mg/dl)	105 (56–144)	11	0,171
IgM *nach* Therapie (mg/dl)	90 (66–104)	11	
IgA *vor* Therapie (mg/dl)	250 (191–349)	11	0,289
IgA *nach* Therapie (mg/dl)	217 (189–300)	11	
Globaffin-Säule	58 %	24	–
Tryptophan-Säule	38 %	24	–
Immunosorba-Säule	17 %	24	–
Wechsel der Säule innerhalb des Behandlungszeitraums	13 %	24	–
IgG-Reduktion (%) Globaffin	−54 (−67 bis −44)	75	<0,001**
IgG-Reduktion (%) Tryptophan	−33 (−35 bis −30)	18	–
IgG-Reduktion (%) Immunosorba	−65 (−72 bis −54)	30	<0,001**
IgM-Reduktion (%) Globaffin	−18 (−26 bis −13)	76	–
IgM-Reduktion (%) Tryptophan	−35 (−38 bis −28)	18	0,002**
IgM-Reduktion (%) Immunosorba	−16 (−40–0)	30	–
IgA-Reduktion (%) Globaffin	−17 (−21 bis −12)	75	–
IgA-Reduktion (%) Tryptophan	−17 (−20 bis −14)	12	–
IgA-Reduktion (%) Immunosorba	−23 (−30 bis −16)	30	0,008 **
Max. RR-Änderung syst. (mm Hg)	−8 (−15–0)	203	<0,001**
Max. RR-Änderung diast. (mm Hg)	0 (−10–0)	203	0,001**

*IQR* „interquartile range“, *Pos.* positiv, *ges.* gesamt, *IA* Immunadsorption, *CCP* zyklisch citrullinierte Peptide, *CRP* C-reaktives Protein, *DMARD* „disease-modifying anti-rheumatic drug“, *Ig* Immunglobulin, *Max.* maximal, *RR* Blutdruck Riva-Rocci, *syst.* systolisch, *diast.* diastolisch

* *p* < 0,05, ** *p* < 0,01

### Indikationen und Abbruchkriterien

**Abb. 2:**
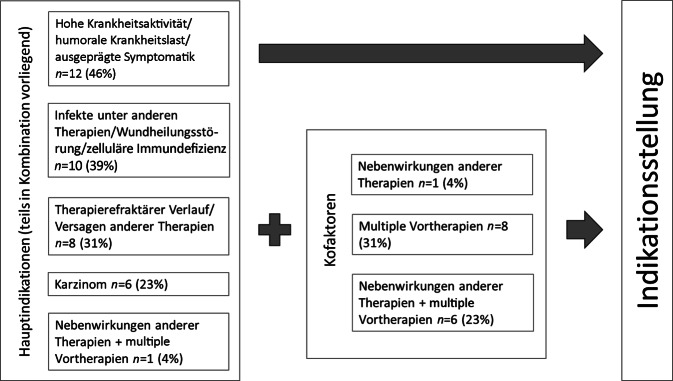
Indikationen für die Immunadsorption

Es konnten 6 Hauptindikationen, oft (69 % der Fälle) mehrere parallel vorliegend, für den Einsatz der Immunadsorption im klinischen Alltag identifiziert werden: Therapieversagen oder Nebenwirkungen anderer Therapien, hohe Krankheitsaktivität bzw. humorale Krankheitslast, Infekte unter anderen Therapien und das Vorliegen eines Karzinoms. Zusätzliche Kofaktoren unterstützten die Indikationsstellung, waren jedoch nie allein ausschlaggebend (Abb. 2).

Die Immunadsorption wurde insgesamt dann eingesetzt, wenn konventionelle Therapien aus unterschiedlichen Gründen gerade keine geeignete Therapieoption darstellten. Die Beendigung erfolgte meist bei Wiederaufnahme einer medikamentösen Immunsuppression (67 %), seltener bei Komplikationen oder unzureichendem Ansprechen (je 28 %). In 68 % der Fälle kam eine begleitende Medikation zum Einsatz, meist Glukokortikoide (65 %) oder Analgetika (65 %), seltener auch eine immunsuppressive Therapie mit DMARDs („disease-modifying anti-rheumatic drugs“) (32 %).

### Sicherheit der Therapie

Bei 25 % der Patient:innen traten Nebenwirkungen unter IA auf, am häufigsten (20 %) ein systolischer Blutdruckabfall <100 mm Hg; in 8 % der Fälle kam es kurz vor Therapieende zu kritischen Abfällen, woraufhin die IA vorzeitig abgebrochen wurde. Die durchschnittliche systolische Blutdruckveränderung betrug −6,89 ± 13,67 mm Hg (*p* ≤ 0,001). Seltene Nebenwirkungen waren Kribbeln im Gesicht, Kopfschmerzen, Schüttelfrost und lokale Schmerzen (jeweils ein Fall). Die IA erfolgte bei 60 % der Patient:innen über einen peripheren, bei 20 % über einen zentralen und bei 20 % der Patient:innen sowohl über einen peripheren als auch einen zentralen Zugang. Bezogen auf alle IA (*n* = 190) entfielen 52 % auf periphere, 48 % auf zentrale Zugänge. Im Untersuchungszeitraum trat eine katheterassoziierte Komplikation mit Explantation bei Verdacht auf Katheterinfektion auf.

### Effektivität

**Abb. 3:**
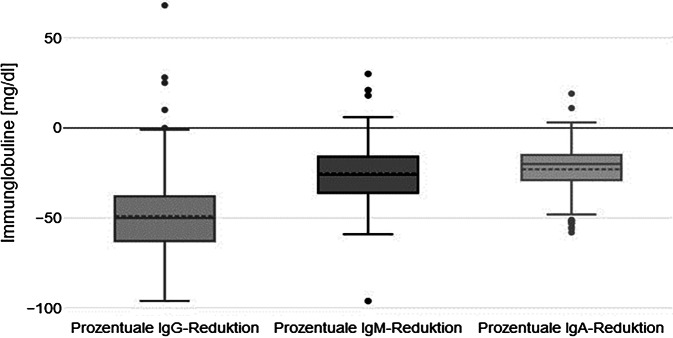
Immunglobulinreduktion durch die Immunadsorption unmittelbar nach der Anwendung. *Ig* Immunglobulin

Zu Beginn der Behandlung zeigten 31 % der Patient:innen erhöhte Ig(Immunglobulin)G-Werte (> 1350 mg/dl) [28]. Die IA führte zu einem signifikanten Rückgang der Immunglobuline (*p* ≤ 0,001): IgG (Median −50 %, IQR: −63 % bis −38 %), IgM (−26 %, IQR: −36 % bis −16 %) und IgA (−20 %, IQR: −29 % bis −15 %) (Abb. 3).

Vor der Therapie mittels Immunadsorption zeigten die Patient:innen höhere Immunglobulinspiegel (IgG: Median = 1810 mg/dl, IQR: 1045 mg/dl–1965 mg/dl; IgM: Median = 105 mg/dl, IQR: 56 mg/dl–144 mg/dl; IgA: Median = 250 mg/dl, IQR: 191 mg/dl–349 mg/dl) als danach (IgG: Median = 1070 mg/dl, IQR: 835 mg/dl–1526 mg/dl; IgM: Median = 90 mg/dl, IQR: 66 mg/dl–104 mg/dl; IgA: Median = 217 mg/dl, IQR: 189 mg/dl–300 mg/dl). Mittels abhängigen t‑Tests konnte gezeigt werden, dass diese Unterschiede für IgG (*p* = 0,007) statistisch signifikant waren. Für IgM und IgA ergab sich kein signifikanter Unterschied, sondern nur ein tendenzieller Abfall (*p* = 0,171 und *p* = 0,289) (Tab. 1).

**Abb. 4:**
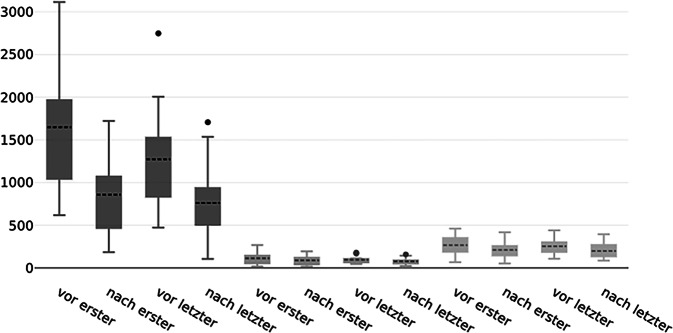
Immunglobulinspiegel vor und nach Immunadsorption (IA) bei der ersten und letzten Behandlung

Der Prozentsatz der Immunglobulinreduktion zwischen der ersten (IgG: Median = −47 %, IQR: −59 % bis −43 %; IgM: Median = −17 %, IQR: −14 % bis −24 %; IgA: Median = −19 %, IQR: −23 %–15 %) und der letzten IA (IgG: Median = −38 %, IQR: −54 % bis −28 %, IgM: Median = −26 %, IQR: −30 % bis −13 %; IgA: Median = −20 %, IQR: −26 %–10 %) zeigte keinen signifikanten Unterschied (Abb. 4 und Tab. 1).

### Adsorber

**Abb. 5:**
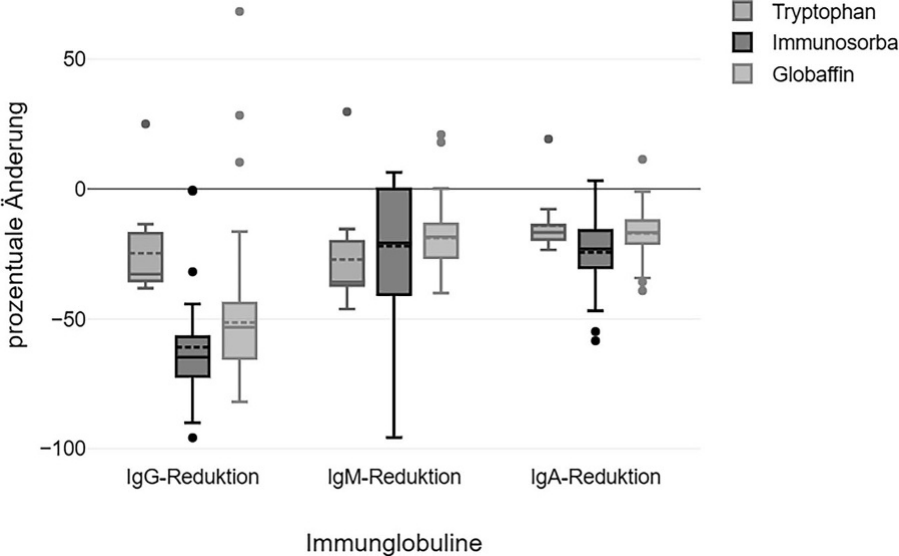
Immunglobulinreduktion bei Verwendung von unterschiedlichen Adsorbern. *Ig* Immunglobulin

Die Untersuchung zeigt signifikante Unterschiede in der Immunglobulinreduktion je nach verwendetem Adsorber (Abb. 5). Globaffin und Immunosorba führten zu einer stärkeren IgG-Reduktion als Tryptophan (*p* ≤ 0,001). Tryptophan bewirkte hingegen die stärkste Reduktion von IgM (*p* = 0,001), während Immunosorba am effektivsten IgA senkte (*p* = 0,008).

### Wirkungen der Immunadsorption

Nach dem ersten Behandlungszyklus sowie über den gesamten Zeitraum zeigten 15 Patient:innen (48 %) einen positiven Therapieeffekt. Vier Patient:innen ohne initiales Ansprechen nach dem ersten Zyklus erhielten erneut IA; 2 von ihnen zeigten später doch einen positiven Therapieeffekt. Zwei von drei Patient:innen mit initialem Ansprechen verloren den positiven Therapieeffekt im weiteren Verlauf.

Die Untersuchung der CRP-Änderung zeigt, dass vor der Therapie mittels IA höhere CRP-Werte vorlagen (1. Zyklus: Median = 2,38 mg/dl, IQR 0,93 mg/dl–4,87 mg/dl; gesamt: Median = 2,38 mg/dl, IQR 0,93 mg/dl–4,87 mg/dl) als nach der Therapie (1. Zyklus: Median = 1,56 mg/dl; IQR 0,56 mg/dl–4,02 mg/dl; gesamt: Median = 1,79 mg/dl; IQR 0,62 mg/dl–4,62 mg/dl). Ein Wilcoxon-Test zeigte, dass dieser Unterschied statistisch nicht signifikant war (1. Zyklus: *p* = 0,269; gesamt: *p* = 0,495) (s. Tab. 1).

Die Patient:innen hatten vor Therapie ebenfalls höhere Glukokortikoiddosen (1. Zyklus: Median = 15 mg; IQR 12,5 mg–35 mg; gesamt: Median = 15 mg; IQR 12,5 mg–35 mg) als nach Therapie (1. Zyklus Median = 12,5 mg; IQR: 10 mg–25 mg; gesamt: Median = 12,5 mg; IQR 10 mg–25 mg). Diese Dosisreduktion war statistisch nicht signifikant (1. Zyklus *p* = 0,715; gesamt: *p* = 0,715) (s. Tab. 1).

In einer Subgruppe von 5 Patient:innen mit RA zeigten sich signifikant verbesserte DAS(„Disease Activity Score“)28-Werte nach dem ersten Zyklus (*p* = 0,015) trotz tendenziell reduzierter Glukokortikoiddosis.

**Abb. 6:**
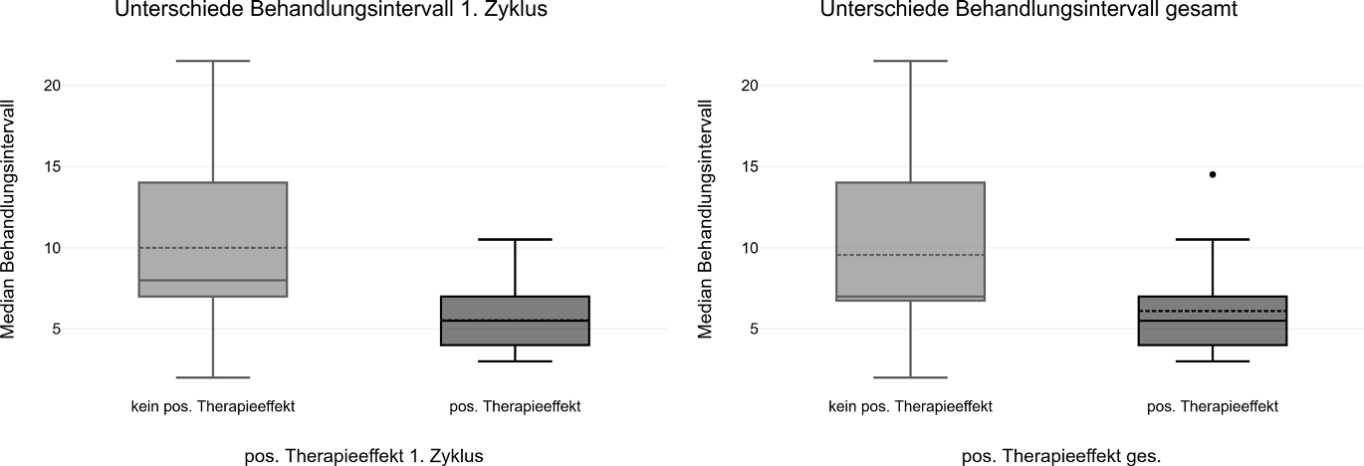
Behandlungsintervalle und positiver (*pos*.) Therapieeffekt 1. Zyklus und gesamter (*ges*.) Behandlungszeitraum

Die Patient:innen mit einem Ansprechen auf die Behandlung erhielten die Immunadsorption mit einer höheren Frequenz und hatten im Median signifikant kürzere Behandlungsintervalle (1. Zyklus: Median = 5,5 Tage; IQR 4 Tage bis 7 Tage; gesamt: Median = 5,5 Tage; IQR 4 Tage bis 7 Tage) als Patient:innen ohne positiven Therapieeffekt (1. Zyklus: Median = 8 Tage; IQR 7 Tage bis 14 Tage; gesamt: Median = 7 Tage; IQR 7 Tage bis 14 Tage) (1. Zyklus: *p* = 0,004; gesamt: *p* = 0,049) (Abb. 6).

### Therapieeffekt und Glukokortikoidtherapie

Unter den Patient:innen mit positivem Therapieeffekt erhielten 60 % Glukokortikoide, 40 % nicht. In der Subgruppe ohne Glukokortikoide (*n* = 11) lagen vor der Therapie mittels IA höhere CRP-Werte vor (1. Zyklus: Median = 3,01 mg/dl, IQR 1,04 mg/dl–7,82 mg/dl; gesamt: Median = 3,01 mg/dl, IQR 1,04 mg/dl–7,82 mg/dl) als nach der Therapie (1. Zyklus: Median = 2,13 mg/dl; IQR 0,32 mg/dl–5,11 mg/dl; gesamt: Median = 2,13 mg/dl; IQR 0,33 mg/dl–5,11 mg/dl). Ein Wilcoxon-Test zeigte, dass dieser Unterschied statistisch nicht signifikant war (1. Zyklus: *p* = 0,407; gesamt: *p* = 0,407).

In dieser Subgruppe konnte bei 55 % der Patient:innen ein positiver Therapieeffekt festgestellt werden – sowohl nach dem ersten Zyklus als auch im gesamten Verlauf.

Ein Chi^2^-Test in der Gesamtpopulation (*n* = 31) zeigte keinen signifikanten Zusammenhang zwischen Glukokortikoidtherapie und Therapieerfolg (*p* = 0,611). Der Behandlungserfolg scheint somit in dieser Kohorte unabhängig vom Einsatz von Glukokortikoiden zu sein.

## Diskussion

Systemisch entzündliche rheumatische Erkrankungen umfassen ein breites Spektrum an akuten und chronischen Manifestationen, die zu Funktionseinschränkungen, Gelenkschäden und einer erhöhten Morbiditäts- und Mortalitätsrate führen [1, 2, 15]. Die IA wird häufig bei akuten Krankheitsschüben eingesetzt, wobei eine hohe Krankheitsaktivität oder humorale Krankheitslast die häufigste Indikation darstellen. Kontraindikationen oder Versagen anderer Therapien spielen ebenfalls eine Rolle. In der Literatur wird die Immunadsorption bei rheumatischen Erkrankungen v. a. als Option bei langwierigen, therapierefraktären Verläufen oder zur Entfernung pathogener Autoantikörper und Immunkomplexen beschrieben, um Krankheitsprogression und Gewebeschäden zu begrenzen [1, 8, 11]. In der bahnbrechenden randomisierten, kontrollierten Studie mit Scheinapherese konnte Felson 1999 einen derart überraschenden Effekt auf die RA-Aktivität zeigen, dass die Verblindung aus ethischen Gründen gestoppt wurde [13]. Die Immunadsorption ist für RA und systemischen Lupus erythematodes (SLE) zugelassen, wird aber auch bei anderen Erkrankungen eingesetzt [16, 17]. Mittlerweile haben wir jedoch ein großes Armamentarium an verschiedenen DMARDs zur Verfügung, sodass die IA bei RA nur noch bei ausgewählten Fällen in Betracht kommt. Dies belegt auch unsere Exploration, die zeigte, dass die IA oft für einen begrenzten Zeitraum als „Bridging-Therapie“ zwischen 2 medikamentösen Therapien eingesetzt wurde. Aufgrund ihres invasiven Charakters und unter Kosten-Nutzen-Abwägungen bei Kosten von ca. 2500 € pro Immunadsorption wird sie nur in seltenen Fällen als Dauertherapie und in der Regel nicht als erste Therapie eingesetzt [14].

Unsere Untersuchung ergab, dass eine höhere Behandlungsfrequenz, z. B. eine IA alle 5 bis 6 Tage, zu einem besseren Ansprechen führen könnte. In der Literatur finden sich noch keine Antworten auf die Frage nach dem optimalen Behandlungsprotokoll für die Immunadsorption [8, 18]. In den Zulassungsstudien der Immunadsorption zur Behandlung der rheumatoiden Arthritis wurde einem festen Behandlungsschema mit einer IA pro Woche über 12 Wochen gefolgt [13]. In der Praxis werden die Behandlungsdauer und Behandlungsanzahl individuell für die Patient:innen festgelegt, bzw. es wird im Verlauf evaluiert, ob die Therapie noch die optimale Therapieoption darstellt [19]. Zur Evaluation eines optimalen Behandlungsprotokolls sind weitere Studien erforderlich.

Durch die Therapie mittels Immunadsorption sinken die Immunglobulinspiegel erwartungsgemäß, im Median um 50 % für IgG, 26 % für IgM und 20 % für IgA mit zusätzlichem Nachweis einer signifikanten Langzeitreduktion von IgG. Diese Reduktionsraten sind von der Tendenz her ähnlich wie in anderen Studien [11, 19–21], somit kann diese Untersuchung die signifikante Immunglobulinreduktion durch die IA bestätigen. Die Immunglobulinsynthese unterliegt keinem Biofeedbackmechanismus (Goldammer et al., 2002); der erneute Anstieg nach Antikörperentfernung ist vermutlich auf einen veränderten Katabolismus und Rückfluss aus dem extravaskulären Raum zurückzuführen [22, 23], sodass keine übermäßige Nachproduktion zu erwarten ist.

Diese Exploration untersuchte den Einsatz von Medikamenten begleitend zur IA. Die Patient:innen erhielten Glukokortikoide (65 %), andere immunsuppressive Therapien (32 %) und Analgetika (65 %). Yamaji et al. (2008) zeigten, dass IA bei SLE eine schnellere und länger anhaltende Reduktion der Steroiddosis als die Kombination von Glukokortikoiden mit einer Cyclophosphamid-Stoßtherapie bewirkt [8, 18]. Auch in unserer Untersuchung war eine Reduktion der Glukokortikoiddosis zu beobachten, diese war jedoch statistisch nicht signifikant – möglicherweise bedingt durch die geringe Fallzahl der entsprechenden Subgruppe.

Der DAS28 verbesserte sich in einer kleinen Subgruppe signifikant. Insgesamt konnte bei 48 % der Patient:innen – sowohl nach dem ersten Zyklus als auch nach der gesamten Therapie – ein positiver Therapieeffekt festgestellt werden. Ein Zusammenhang mit einer begleitenden Glukokortikoidtherapie bestand dabei nicht. Prinzipiell kann also eine Wirksamkeit der Immunadsorption angenommen werden.

Ein positiver Therapieeffekt zeigte sich in einem Fall auch erst nach einem zweiten Zyklus, obwohl der erste keine Wirkung zeigte – im Gegensatz zu Felson et al. (1999), die in solchen Fällen keinen späteren Effekt fanden [12, 13]. Unsere Ergebnisse widersprechen dieser Einschätzung zumindest teilweise.

Im Vergleich zur Literatur liegt unsere Erfolgsquote höher als in den Zulassungsstudien mit Erfolgsraten von 41,7 und 31,9 %, was durch Unterschiede in Indikationsstellung und Begleittherapie erklärbar ist [12, 13]. Fuchs et al. (2022) berichteten bei anderen Erkrankungen sogar eine Ansprechrate von 63 % [19]. Dies unterstreicht die Bedeutung der Patientenselektion und Grunderkrankung für den Therapieerfolg.

In dieser Untersuchung zeigten sich signifikante Unterschiede in der Reduktion einzelner Immunglobulinklassen durch verschiedene Adsorber: Globaffin führte zur stärksten IgG-Reduktion, Immunosorba reduzierte sowohl IgG als auch IgA am effektivsten, während Tryptophan IgM am stärksten senkte. Diese Unterschiede bieten einen interessanten Ansatzpunkt für weitere Untersuchungen. Die gezielte Auswahl des Adsorbers bei verschiedenen Krankheitsbildern, wie z. B. IgG-vermittelten Autoimmunerkrankungen, bei Patient:innen mit Sicca-Symptomatik oder erosiver RA mit erhöhten Leveln an IgA-RF (Rheumafaktor), entsprechend der immunologischen Konstellation, könnte zukünftig zur Optimierung der Therapie beitragen [24, 25]. Hier sind jedoch weitere Untersuchungen nötig, um zu evaluieren, ob die Auswahl eines spezifischen Adsorbers unter Berücksichtigung der Unterschiede der Adsorber auch einen Effekt auf den Therapieerfolg hat.

Die IA war in dieser Untersuchung insgesamt gut verträglich. Bei 25 % der Patient:innen traten Nebenwirkungen wie Blutdruckabfall, Parästhesien oder Kopfschmerzen auf; schwerwiegende Nebenwirkungen waren selten (kritischer Blutdruckabfall in 3 Fällen, 8 %). Im Vergleich zu anderen Studien mit Nebenwirkungsraten von bis zu 88 % wurden relativ wenige Nebenwirkungen beobachtet [12–14]. Die Rate an schwerwiegenden Nebenwirkungen der IA wird jedoch auch in der Literatur teilweise, z. B. in dem Paper von Matic et al. (2001) mit 1 % bei Verwendung der Immunosorba-Säule, als sehr gering beschrieben [16]. Insgesamt ist die Varianz der Nebenwirkungsraten hoch, was sich unter anderem durch Unterschiede in den Patient:innenkollektiven und Grunderkrankungen erklären lässt.

Die Ergebnisse dieser Analyse basieren auf retrospektiven Daten aus dem klinischen Alltag und zeigen Heterogenitäten sowohl in Bezug auf die Zeiträume der IA-Behandlungen als auch auf die eingeschlossenen Krankheitsbilder. Viele Auswertungen waren daher nur in kleinen Subgruppen möglich. Zudem war keine einheitliche Definition des Therapieerfolgs möglich, da verschiedene Erkrankungen mit unterschiedlichen klinischen Parametern eingeschlossen wurden. Eine weitere Einschränkung bestand darin, dass in einigen Fällen zusätzlich eine Glukokortikoidtherapie verabreicht wurde. Jedoch konnte eine Glukokortikoidreduktion beobachtet werden, und auch bei Patient:innen ohne begleitende Steroidgabe traten positive Therapieeffekte auf.

Da insgesamt jedoch nur wenige Studien zum Thema Immunadsorption bei rheumatischen Erkrankungen existieren, kann diese Exploration insbesondere auch bei der Planung weiterer Untersuchungen im klinischen Alltag einen Beitrag leisten. Zur Evaluation der Ergebnisse sollten daher weitere, kontrollierte Untersuchungen mit höheren Patientenzahlen angestrebt werden.

Die Immunadsorption hat das Potenzial, eine Remission herbeizuführen oder die Symptome mit wenigen Nebenwirkungen zu kontrollieren [8]. Sie birgt großes Potenzial, insbesondere für Patient:innen, die auf konventionelle Therapien unzureichend ansprechen oder diese nicht vertragen. Weitere Studien könnten die Indikationen erweitern und prädiktive Marker für den Therapieerfolg ermitteln [1, 14]. Die Immunadsorption, möglicherweise in Kombination mit Biologika, bietet neue Optionen für die Behandlung von Autoimmunkrankheiten [1, 26–29]. Ein vielversprechender Ansatz bei der Behandlung vieler Erkrankungen ist die individualisierte Medizin. Auch im Bereich der Rheumatologie gibt es bei der medikamentösen Immuntherapie viele Fortschritte, jedoch geht diese Immuntherapie zum Teil auch mit schweren Nebenwirkungen und Toxizität einher. Viele Patient:innen erreichen trotz dieser hochmodernen Therapie keine langfristige Remission [30]. Es ist daher wichtig, die Therapie auf die spezifischen Bedürfnisse der Patient:innen abzustimmen.

## Fazit für die Praxis

- Die Immunadsorption (IA) stellt eine gut verträgliche und sichere Therapieoption dar, die insbesondere bei schweren rheumatologischen Erkrankungen sowie bei relevanten Komorbiditäten eingesetzt wird – häufig in Kombination mit medikamentösen Therapien. In solchen Fällen dient sie nicht selten als sog. „Bridging-Therapie“, bis eine krankheitsmodifizierende Behandlung greift oder etabliert werden kann.
- Insbesondere in komplexen oder therapierefraktären Verläufen kann die IA auch als Ultima Ratio zum Einsatz kommen. Sie führt häufig zu einer signifikanten Reduktion zirkulierender Immunglobuline und Autoantikörper, was mit einer klinischen Besserung einhergehen kann.
- Die IA kann somit eine wertvolle therapeutische Option im Rahmen individualisierter Behandlungsstrategien darstellen und sollte in zukünftigen Studien weiter untersucht werden.

## Data Availability

Die in dieser Studie erhobenen Datensätze können auf begründete Anfrage beim Korrespondenzautor angefordert werden.

## References

[1] Bambauer R, Latza R, Bambauer C, Burgard D, Schiel R (2013) Therapeutic apheresis in autoimmune diseases. Open Access Rheumatol 5:93–103. 10.2147/OARRR.S34616 27790028 10.2147/OARRR.S34616 PMC5074795

[2] Bakker MF, Jacobs JWG, Verstappen SMM, Bijlsma JWJ (2007) Tight control in the treatment of rheumatoid arthritis: efficacy and feasibility. Ann Rheum Dis 66(3):56–60. 10.1136/ard.2007.078360 10.1136/ard.2007.078360 PMC2095293 17934098

[3] Mitratza M, Klijs B, Hak AE, Kardaun JWPF, Kunst AE (2021) Systemic autoimmune disease as a cause of death: mortality burden and comorbidities. Rheumatology 60:1321–1330. 10.1093/rheumatology/keaa537 32944773 10.1093/rheumatology/keaa537 PMC7937014

[4] WHO Rheumatoid arthritis. https://www.who.int/news-room/fact-sheets/detail/Rheumatoid-arthritis (Erstellt: 28. Juli 2023). Zugegriffen: 21. Sept. 2023

[5] AWMF S2e-Leitlinie Therapie der rheumatoiden Arthritis mit krankheitsmodifizierenden Medikamenten. https://register.awmf.org/de/leitlinien/detail/060-004 (Erstellt: 1. Apr. 2018). Zugegriffen: 21. Sept. 2023 10.1007/s00393-018-0481-y 29968101

[6] Nagy G, Roodenrijs NMT, Welsing PMJ, Kedves M, Hamar A, van der Goes MC et al (2022) EULAR points to consider for the management of difficult-to-treat rheumatoid arthritis. Ann Rheum Dis 81:20–33. 10.1136/annrheumdis-2021-220973 34407926 10.1136/annrheumdis-2021-220973 PMC8761998

[7] Albrecht. S1-Leitlinie: Medikamentösen Therapie der rheumatoiden Arthritis

[8] Yamaji K (2017) Immunoadsorption for collagen and rheumatic diseases. Transfus Apher Sci 56:666–670. 10.1016/j.transci.2017.08.012 28970002 10.1016/j.transci.2017.08.012

[9] Chen AY, Wolchok JD, Bass AR (2021) TNF in the era of immune checkpoint inhibitors: friend or foe? Nat Rev Rheumatol 17:213–223. 10.1038/s41584-021-00584-4 33686279 10.1038/s41584-021-00584-4 PMC8366509

[10] Labrosse R, Haddad E (2023) Immunodeficiency secondary to biologics. J Allergy Clin Immunol 151:686–690. 10.1016/j.jaci.2023.01.012 36706964 10.1016/j.jaci.2023.01.012

[11] Braun N, Bosch T (2000) Immunoadsorption, current status and future developments. Expert Opin Investig Drugs 9:2017–2038. 10.1517/13543784.9.9.2017 11060790 10.1517/13543784.9.9.2017

[12] Gendreau RM (2001) A randomized double-blind sham-controlled trial of the Prosorba column for treatment of refractory rheumatoid arthritis. Ther Apher 5:79–83 11354303

[13] Felson DT, LaValley MP, Baldassare AR, Block JA, Caldwell JR, Cannon GW et al (1999) The Prosorba column for treatment of refractory rheumatoid arthritis: a randomized, double-blind, sham-controlled trial. Arthritis Rheum 42(10):2153–2159. 10.1002/1529-0131 (〈2153::AID-ANR16〉3.0.CO;2‑W.) 10524687 10.1002/1529-0131(199910)42:10<2153::AID-ANR16>3.0.CO;2-W

[14] Poullin P, Announ N, Mugnier B, Guis S, Roudier J, Lefèvre P (2005) Protein A-immunoadsorption (Prosorba column) in the treatment of rheumatoid arthritis. Joint Bone Spine 72:101–103. 10.1016/j.jbspin.2004.02.009 15797486 10.1016/j.jbspin.2004.02.009

[15] Mitratza M, Klijs B, Hak AE, Kardaun JWPF, Kunst AE (2021) Systemic autoimmune disease as a cause of death: mortality burden and comorbidities. Rheumatology 60:1321–1330. 10.1093/rheumatology/keaa537 32944773 10.1093/rheumatology/keaa537 PMC7937014

[16] Matic G, Bosch T, Ramlow W (2001) Background and indications for protein A‑based extracorporeal immunoadsorption. Ther Apher 5:394–403 11778926 10.1046/j.1526-0968.2001.00370.x

[17] Kassenärztliche Bundesvereinigung. Therapeutische Hämapheresen (selektive Verfahren mit Plasmadifferentialtrennung). https://www.g-ba.de/downloads/40-268-240/HTA-Apheresen.pdf (Erstellt: 25. Juli 2003). Zugegriffen: 17. Dez. 2023

[18] Sugimoto K, Yamaji K, Yang K‑S, Kanai Y, Tsuda H, Hashimoto H (2006) Immunoadsorption plasmapheresis using a phenylalanine column as an effective treatment for lupus nephritis. Ther Apher Dial 10:187–192. 10.1111/j.1744-9987.2006.00362.x 16684222 10.1111/j.1744-9987.2006.00362.x

[19] Fuchs K, Rummler S, Ries W, Helmschrott M, Selbach J, Ernst F et al (2022) Performance, clinical effectiveness, and safety of immunoadsorption in a wide range of indications. Ther Apher Dial 26:229–241. 10.1111/1744-9987.13663 33914397 10.1111/1744-9987.13663 PMC9291474

[20] Belàk M, Borberg H, Jimenez C, Oette K (1994) Technical and clinical experience with protein A immunoadsorption columns. Transfus Sci 15:419–422. 10.1016/0955-3886(94)90174-0 10155559 10.1016/0955-3886(94)90174-0

[21] Biesenbach P, Schmaldienst S, Smolen JS, Hörl WH, Derfler K, Stummvoll GH (2009) Immunoadsorption in SLE: three different high affinity columns are adequately effective in removing autoantibodies and controlling disease activity. Atheroscler Suppl 10:114–121. 10.1016/S1567-5688(09)71824-0 20129388 10.1016/S1567-5688(09)71824-0

[22] Goldammer A, Derfler K, Herkner K, Bradwell AR, Hörl WH, Haas M (2002) Influence of plasma immunoglobulin level on antibody synthesis. Blood 100:353–355. 10.1182/blood-2002-01-0128 12070050 10.1182/blood-2002-01-0128

[23] Blandino R, Secreted IgM BN (2019) New tricks for an old molecule. J Leukoc Biol 106:1021–1034. 10.1002/JLB.3RI0519-161R 31302940 10.1002/JLB.3RI0519-161R PMC6803036

[24] Jorgensen C, Anaya JM, Cognot C, Sany J (1992) Rheumatoid arthritis associated with high levels of immunoglobulin a: clinical and biological characteristics. Clin Exp Rheumatol 10:571–575 1483308

[25] Jorgensen C, Legouffe MC, Bologna C, Brochier J, Sany J (1996) IgA isotype rheumatoid factor in rheumatoid arthritis: clinical implications. Clin Exp Rheumatol 14:301–304 8809445

[26] Bambauer R, Schwarze U, Schiel R (2000) Cyclosporin A and therapeutic plasma exchange in the treatment of severe systemic lupus erythematosus. Artif Organs 24:852–856. 10.1046/j.1525-1594.2000.06623.x 11119071 10.1046/j.1525-1594.2000.06623.x

[27] Schiel R, Bambauer R, Latza R, Klinkmann J (1997) Cyclosporine and therapeutic plasma exchange in treatment of progressive autoimmune diseases. Artif Organs 21:983–988. 10.1111/j.1525-1594.1997.tb00512.x 9288868 10.1111/j.1525-1594.1997.tb00512.x

[28] Gutschmidt HJ, Euler HH, Albrecht J, Asbeck F, Kleine LH, von Klinggräff C, Löffler H (1986) Cyclophosphamid-Stosstherapie synchronisiert mit Plasmapheresen bei rapid progredienter Glomerulonephritis. Dtsch Med Wochenschr 111:1439–1444. 10.1055/s-2008-1068648 ([Cyclophosphamide stosstherapy synchronized with plasmapheresis in rapidly progressive glomerulonephritis]) 3757801 10.1055/s-2008-1068648

[29] Dau PC, Callahan J, Parker R, Golbus J (1991) Immunologic effects of plasmapheresis synchronized with pulse cyclophosphamide in systemic lupus erythematosus. J Rheumatol 18:270–276 1827161

[30] Bhamidipati K, Wei K (2022) Precision medicine in rheumatoid arthritis. Best Pract Res Clin Rheumatol 36:101742. 10.1016/j.berh.2022.101742 35248489 10.1016/j.berh.2022.101742 PMC8977251

